# Growth at the limits: comparing trace metal limitation of a freshwater cyanobacterium (*Dolichospermum lemmermannii*) and a freshwater diatom (*Fragilaria crotonensis*)

**DOI:** 10.1038/s41598-021-04533-9

**Published:** 2022-01-10

**Authors:** Markus Dengg, Claudine H. Stirling, Malcolm R. Reid, Piet Verburg, Evelyn Armstrong, Laura T. Kelly, Susanna A. Wood

**Affiliations:** 1 Department of Geology and Centre for Trace Element Analysis, University of Otago, Dunedin, 9016 New Zealand; 2 National Institute of Water and Atmospheric Research (NIWA), Hamilton, 3251 New Zealand; 3 Department of Marine Science, NIWA/University of Otago Research Centre for Oceanography, University of Otago, Dunedin, 9010 New Zealand; 4 Cawthron Institute, Nelson, 7010 New Zealand

**Keywords:** Freshwater ecology, Ecology

## Abstract

Freshwater phytoplankton blooms are increasing in prevalence and there are conflicting views on whether trace metals limit growth of key species and thus bloom formation. The Taupō Volcanic Zone (TVZ), New Zealand, was formed by multiple eruptions of a super-volcano which emitted rhyolitic tephra leaving lakes depleted in trace metals. This provides an opportunity to test the potential of trace metal limitation on freshwater phytoplankton growth under nanomolar concentrations. Growth responses of two algal species isolated from Lake Taupō, *Dolichospermum lemmermannii* (cyanobacteria) and *Fragilaria crotonensis* (diatom), to six biologically important trace metals (manganese, iron, zinc, cobalt, copper and molybdenum) were examined in culture experiments. These were conducted at three trace metal concentrations: (1) ambient, (2) two-times ambient, and (3) ten-times ambient concentrations in Lake Taupō. Elevated concentrations of iron significantly increased growth rates and maximum cell densities in *D. lemmermannii,* whereas no significant concentration dependence was observed for other trace metals. *Fragilaria crotonensis* showed no significant growth response to elevated concentrations of trace metals. These results highlight the importance of iron as a growth limiting nutrient for cyanobacteria and indicate that even small (twofold) increases in Fe concentrations could enhance cyanobacteria growth rates in Lake Taupō, potentially causing cyanobacterial blooms.

## Introduction

Increasing anthropogenic pressures, such as the use of fertilizers to support agricultural practices, alterations in water flow regimes via the implementation of dam systems, and land use intensification through accelerated urbanisation^1^ are decreasing the health of freshwater systems globally^2^. As water bodies become more eutrophic and climates continue to warm^3,4^, water quality is declining and ‘cyanobacterial harmful algae blooms’ (CHABs) are becoming increasingly prevalent in freshwater systems^5^. The ability of cyanobacteria to produce toxins, such as microcystins and anatoxins^6,7^, which are harmful to aquatic life and potentially dangerous for humans^8^, have led to the suggestion that CHABS now pose “the greatest inland water quality threat to public health and aquatic ecosystems”^9^.

Over the past five decades, studies have focused on identifying variables that enhance the formation of CHABs in order to better understand drivers and take preventative action. Results showed that increases in the levels of the macronutrients nitrogen (N) or phosphorus (P), or both, contribute the most to the formation of CHABs^10,11^. However, as many species responsible for CHAB formation are capable of atmospheric N-fixation via the nitrogenase enzymes^12^, there is ongoing debate about the relative importance of N versus P in regulating CHAB formation^13–16^. Trace metal micronutrients, such as manganese (Mn), iron (Fe), zinc (Zn), cobalt (Co), copper (Cu) and molybdenum (Mo), although often present in trace quantities in the environment, are required for enzymatic processes in phytoplankton.

Iron and Mn are required in the highest quantities as they are both important components of the central electron acceptor proteins, enabling carbon dioxide (CO_2_) fixation^17^. Iron also plays an important role in N fixation by cyanobacteria^18^ as it forms, together with molybdenum (Mo), part of the dinitrogenase subunit in cells, which allows the fixation of N from its gaseous form (N_2_) into bioavailable forms of N^19,20^. Other trace metals also play essential roles in cell physiology. For example, Zn is a critical component of the RUBISCO (Ribulose-1,5-bisphosphate carboxylase/oxygenase) enzyme, which catalyses the first step of CO_2_-fixation^21,22^. Additionally, Zn forms a component of the enzymes needed for the DNA/RNA polymerase replication and transcription, and has a structural role in several other enzymes, and in the immune responses of cells^22–25^. Cobalt has an important function as the catalytic centre of Vitamin B12^26^ and can replace Zn in enzymes and pathways under Zn-limiting conditions^27^. Copper forms part of the electron transport proteins ‘cytochrome c’ and ‘plastocyanin’, enabling electron transport across cell membranes, and is crucial for the function of several ‘oxidases’, such as ascorbate oxidase^25,28^.

The lakes in the North Island of New Zealand, situated in the Taupō Volcanic Zone (TVZ), extending from Mount Ruapehu in the south to White Island in the north (Supplementary Fig. S1, online), are influenced by ash deposits from past volcanic activity. Eruptive events have covered the TVZ and lakebeds across the region with rhyolitic tephra, which has 20-fold lower trace metal concentrations than many other soils^29^. Accordingly, the concentrations of some trace metals, such as Co, in Lake Taupō waters that have interacted with both TVZ soils and lakebed sediments are 1000-fold lower than those observed in other lakes worldwide (Dengg et al., unpublished data). Therefore, the TVZ lakes and the phytoplankton residing in their waters are uniquely suited to study the effect of trace metal limitation on the formation of CHABs due to their naturally low trace metal concentrations and growth requirements.

Several studies conducted to date have shown the potential for trace metals to limit phytoplankton growth in freshwater lakes^30–33^ with Fe identified as the trace metal most often limiting growth^34,35^ and CHAB formation^19,36,37^. However, further research is required to clarify the role of trace metals in CHAB formation and to distinguish concentration thresholds that result in enhanced growth of cyanobacteria and other algal taxa, such as diatoms (siliceous phytoplankton). This study aims to compare the growth responses of the cyanobacteria species *Dolichospermum lemmermannii* (Brébisson ex Bornet & Flahault P. Wacklin, L. Hoffmann & J. Komárek) and the diatom species *Fragilaria crotonensis* (Kitton) under differing low-level trace metal concentrations, at the sub-nanomolar level, but with constant levels of nitrogen and phosphorous. While a previous investigation of two cyanobacteria species isolated from Lake Taupō waters showed that increased concentrations of Fe, Co and Mo positively affected growth rates and maximum cell concentrations^36^, the effect of elevated trace metal concentrations on diatom growth was not tested. Therefore, we extend this investigation of Lake Taupō phytoplankton species by exploring differences in the growth responses of both cyanobacteria and diatoms to varying availability of trace metal micronutrients under 10 to 100-fold lower trace metal concentrations than tested previously*. Dolichospermum lemmermannii* is a cyanobacteria that forms CHABs in other freshwater lakes^13,38,39^ and has the potential to form blooms in Lake Taupō as well. *Fragilaria crotonensis* is found in high abundance in Lake Taupō, dominating algal assemblages during times of lake mixing from autumn until spring when algal biomass is highest in Lake Taupō^40,41^. Our study had two key hypotheses. First, we proposed that increased concentrations of trace metals would yield positive growth responses from both species. However, the cyanobacteria species was expected to show a stronger growth response than the diatom species due to the difference in the trace metal demand, related to (i) the difference in cell size of 45 × 3 × 3 µm for pennate cells of *F. crotonensis* compared to 5–20 µm diameter for spherical cells of *D. lemmermannii*^42,43^ and (ii) the ability of cyanobacteria to access trace metals via metallophores^44^, allowing for enhanced trace metal uptake. Second, we theorised that increased concentrations of Fe would yield stronger growth responses than the other trace metals investigated, as Fe is the trace element of highest demand in cell biochemistry^17^. Confirmation of our hypotheses through the results of this study, would indicate that increasing trace metal concentrations would likely lead to enhanced phytoplankton growth and CHAB formation in lakes containing low trace metal concentrations.

To test these hypotheses, the growth responses of *D. lemmermannii* and *F. crotonensis* to the biologically important trace metals Mn, Fe, Co, Cu, Zn and Mo were investigated in a laboratory growth experiment*.* Annual averaged Lake Taupō dissolved trace metal concentrations were selected as the ambient conditions for this experiment and concentrations of 2 × and 10 × ambient were tested for growth response. Whilst most growth experiments conducted with freshwater phytoplankton have been performed with mid- to high concentrations of trace metals in the micromolar range^30,36,45^, we have made a concerted effort to undertake the experiments at the naturally low trace metal concentrations of Lake Taupō, which are in the nanomolar range or lower.

## Methods

Conducting growth experiments with low trace metal concentrations in the sub-nanomolar range comes with great challenges^46^ and requires careful planning with multiple laboratory controls in place. In the present study, media and sample preparation were performed in a Class 100 (ISO 5) clean room within a Class 10 (ISO 4) laminar flow bench at the Centre for Trace Element Analysis, University of Otago (New Zealand) using ultra high-purity reagents, either purified in-house using sub-boiling distillation or purchased commercially, and trace metal clean working procedures at all times^46^. Furthermore, the trace metal concentrations of the media during all preparation steps, as well as at the beginning and end of the experiment, were measured to assess the extent of any inadvertent contamination of the growth media.

### Experimental species

Unialgal cultures of *D. lemmermannii* (CAWBG680) and *F. crotonensis* (CAWB503) were obtained from the Cawthron Institute Culture Collection of Microalgae (New Zealand)^47^. Both species were originally isolated from Lake Taupō from samples collected between February 2018 and June 2019^36^.

### Experimental design

The effects on phytoplankton growth by Mn, Fe, Co, Cu, Zn and Mo supplied at ambient Lake Taupō concentrations (annually averaged) of 2.3, 22.1, 0.072, 1.4, 0.55 and 2.3 nmol L^−1^ respectively, as well as two times ambient levels (4.6, 44.2, 0.14, 2.8, 1.1 and 4.6 nmol L^−1^, respectively) and ten times ambient concentrations (23, 221, 0.72, 14, 5.5 and 23 nmol L^−1^, respectively) were investigated. A mix of all trace metals was used to prepare the base medium at ambient metal concentrations, and then each treatment was spiked individually with the trace metal to attain the required elevated concentrations.

### Media preparation

Modified MLA medium^48^, prepared using macronutrient stock solutions of MgSO_4_.7H_2_O, NaNO_3_, K_2_HPO_4_, H_3_BO_3_, H_2_SeO_3_, admixed with a vitamin stock solution containing Biotin, Vitamin B_12_, Thiamine HCl, and spiked with the trace metals of interest, prepared in 12.5 mM ethylenediaminetetraacetic acid (EDTA), at ambient Lake Taupō concentrations was used as the base growth medium and as treatment ‘Mix 1x’ (Table 1). To facilitate diatom growth, silicon (Si), in the form of Na_2_Si, was added to the medium.

**Table 1:** Experimental set up.

Treatment	Mn	Fe	Co	Cu	Zn	Mo
Mix 1 ×	2.25 (0.03)	25.6 (2.7)	0.085 (0.006)	1.7 (0.2)	< LOD	2.6 (0.2)
Mix 2 ×	2 × Mix 1 ×	2 × Mix 1 ×	2 × Mix 1 ×	2 × Mix 1 ×	2 × Mix 1 ×	2 × Mix 1 ×
Mix 10 ×	10 × Mix 1 ×	10 × Mix 1 ×	10 × Mix 1 ×	10 × Mix 1 ×	10 × Mix 1 ×	10 × Mix 1 ×
Mn 2 ×	2 × Mix 1 ×					
Mn 10 ×	10 × Mix 1 ×					
Fe 2 ×		2 × Mix 1 ×				
Fe 10 ×		10 × Mix 1 ×				
Co 2 ×			2 × Mix 1 ×			
Co 10 ×			10 × Mix 1 ×			
Cu 2 ×				2 × Mix 1 ×		
Cu 10 ×				10 × Mix 1 ×		
Zn 2 ×					2 × Mix 1 ×	
Zn 10 ×					10 × Mix 1 ×	
Mo 2 ×						2 × Mix 1 ×
Mo 10 ×						10 × Mix 1 ×

Trace metal concentrations for manganese (Mn), iron (Fe), cobalt (Co), copper (Cu), zinc (Zn) and molybdenum (Mo) measured (nmol*L^−1^) of treatment Mix 1 × and expected concentrations for each other treatment tested. Mix 1 × refers to the concentration of the respective trace metal present in the base media. Every treatment was prepared in a base of Mix 1 × media. ± 1SD of measurements are given in brackets.

To remove pre-existing trace metal contaminants, each of the macronutrient stock solutions were passed through an ion-exchange, rigid methacrylic polymer resin (Toyopearl, Merck, NZ), which binds trace metals but allows other nutrients to pass through. Final concentrations of macronutrients in the base medium were 0.2 mmol L^−1^ for MgSO_4_·7H_2_O, 2.0 mmol L^−1^ for NaNO_3_, 0.4 mmol L^−1^ for K_2_HPO_4_, 0.04 mmol L^−1^ for H_3_BO_3_, 0.01 mmol L^−1^ for H_2_SeO_3_ and 0.204 mmol L^−1^ for Na_2_Si. To reduce the risk of potential additional trace metal contamination from the vitamin stock solution, only half of the typical concentration (diluted v/v) of the vitamin solution was added when the MLA medium was prepared. To avoid biological contamination, the MLA media was passed through a 0.22 µm filter (Sartobran, Sartorius, Germany), before the addition of trace metals, to remove any microorganisms potentially present in the media stock solutions or introduced during media preparation.

The trace metal content of the purified nutrient stock solutions and the mixed MLA medium before the addition of trace metals, was measured by quadrupole inductively coupled plasma mass spectrometry (Q-ICP-MS) to investigate the extent of possible trace metal contamination during media preparation. For all the trace metals of interest, the concentrations were determined to be below the instrumental ‘limit of detection’ (LOD) for the respective elements (see ‘Trace metal concentration determination’). Each treatment (Table 1) was then spiked as required with trace elements, to reach target concentrations of two times and ten times the ambient concentrations in Lake Taupō for the ‘2 × ’ and ‘10 × ’ treatments, respectively, and accuracy of the trace metal addition was validated by Q-ICP-MS measurements.

### Estimation of the bioavailable fraction

Modelling of the bioavailable fraction of trace metals in the growth media was performed prior to the experiments using Visual MINTEQ (vers. 3.1, J.P. Gustafsson, 2013). This allows the equilibrium mass distribution between solid phases, dissolved species, and adsorbed species to be calculated under different environmental conditions. The modelling results were used to estimate the bioavailability of trace metals, ensuring trace metal bioavailability in culture media reaches levels similar to those observed in natural freshwaters^37,49^. The model input parameters included temperature (°C), pH, major ion and trace metal concentrations (µmol L^−1^), as well as the concentration of EDTA (µmol L^−1^) used as a ligand in the experiments.

### Culture preparation

Prior to the start of the experiment, cultures of *D. lemmermanii* and *F. crotonensis* were grown to a high density in a standard MLA medium, not chemically modified to attain low trace metal concentrations. Growth conditions were 17.5 °C (± 0.5 °C) on a 12 h:12 h light–dark cycle with an irradiance of 125 ± 30 µmol photons·m^−2^ s^−1^. To reduce the transfer of remnant trace metals present in the culture media and cells to the experimental treatments, the cultures were filtered through a nylon plankton net mesh (11 µm) and washed with trace metal free MLA medium. The washed stock cultures were gently transferred from the mesh into 100 mL polypropylene Digitubes (SCP Science, NZ) and grown in modified MLA medium, containing half the ambient concentration of the tested trace metals, for 1 week (3–7 generations), to allow the consumption of any intracellularly stored trace metals prior to the start of the experiments.

An aliquot (2 mL) of each stock culture was pipetted separately into Digitubes containing aliquots (98 mL) of the respective treatments (Table 1). Each trace metal treatment was tested in triplicate. The culture containers were incubated at the temperature and light conditions described above for 18 and 11 days for *D. lemmermannii* and *F. crotonensis*, respectively.

### Sample preparation for trace metal concentration analysis

Immediately after the addition of the phytoplankton stock cultures to the different treatments, subsamples (2 mL) were pipetted from each treatment for later assessment of trace metal concentrations via Q-ICP-MS. These subsamples were filtered (0.22 µM, Millex-GV filter, Merck, NZ) then admixed with 14 M HNO_3_ (20 µL). Additional subsamples (2 mL) were also taken at the end of the experiment to investigate trace metal uptake by phytoplankton and to account for any trace metal contamination that may have been introduced by the sub-sampling of cultures for growth measurements during the experiment. All subsamples were stored at room temperature prior to trace metal concentration analysis.

### Subsampling of cultures for cell enumeration and estimation of growth phases

Each vial containing cultures was gently mixed before subsampling, and subsamples (3 mL) were collected on days 0, 3, 6, 8, 10, 12, 14, 16, and 18, and days 0, 3, 5, 7 and 11 for *D. lemmermannii* and *F. crotonensis,* respectively. From each subsample, a 1 mL aliquot was taken and immediately preserved with Lugol’s iodine for later cell enumeration. The phycocyanin and chlorophyll a (Chl *a*) content of the cultures was measured using a CyanoFluor fluorometer (Turner Designs, USA). CyanoFluor relative fluorescence was converted to µg L^−1^ for phycocyanin and *Chl a* using in-house established conversion factors^36^ (pers. comm. Jonathan Puddick). The data obtained from fluorescence measurements via CyanoFluor was then used to (i) detect the lag, as well as the exponential and stationary growth phase for each culture, and (ii) select samples for further cell enumeration using microscopy to determine the maximum cell densities and maximum growth rate of each culture (Supplementary Fig. S2, online).

### Cell enumeration

The concentration of cells in selected samples was determined from the Lugol’s preserved sample. A subsample (up to 1 mL, adjusted based on cell concentration) was pipetted into a 12-well plate (Costar, Corning Incorporated, ME, USA) and allowed to settle for approximately 1.5 h. Cells were enumerated using an inverted microscope (CKX41, Olympus or Axiovert 25, Zeiss). Enumeration was undertaken either by counting all cells in a settling chamber or by counting transects, depending on the cell density. Subsequently, maximum growth rates were calculated using Eq. (1):

1 $${\mathrm{Growth\,rate }\,(\mathrm{day }}^{-1})=Ln\frac{(\mathrm{C}1/\mathrm{C}2)}{(\mathrm{T}2-\mathrm{T}1)}$$

where C1 is the cell concentration at time T1, and C2 is the cell concentration at time T2, with times given in days.

### Trace metal concentration determination

Samples were filtered, acidified and measured directly, without further pre-concentration or dilution using Q-ICP-MS and a 7900 instrument (Agilent Technologies, USA) located at the Centre for Trace Element Analysis, University of Otago. The concentrations of the elements of interest were determined from measurements of the isotopes 55-Mn, 56-Fe, 59-Co, 60-Ni, 63-Cu, 66-Zn and 95-Mo to avoid isobaric and polyatomic interferences that overlap in mass. An instrument calibration curve using a series of multi-element standards containing a suite of elements of known concentration with at least three calibration points was performed at the beginning of the analysis. An internal standard containing seven elements (Supplementary Information S3, online) was admixed to every sample to correct for instrumental drift and matrix effects. The performance of the analysis was assessed based on the repeat measurement of the matrix-matched river water certified reference material, SLRS5 (National Research Council, Canada). The mean values are in very good agreement, within error, with accepted values for all investigated elements. For each sample, trace metal concentrations are reported in nmol L^−1^ with their associated 1SD uncertainties of ± 6% for all elements. Limit of detection values, based on repeat measurement (n = 7) of a low concentration standard were of 0.18 for Mn, 3.58 for Fe, 0.03 for Co, 0.16 for Cu, 0.76 for Zn and 0.21 nmol L^−1^ for Mo, while the limits of quantification (LOQ) were calculated as three times the respective LOD values.

Additionally, ten ‘zero-blanks’ comprising high-purity water, and two ‘medium-blanks’ derived of MLA medium without the addition of trace metals, were processed throughout the growth experiments and were subjected to the same handling procedures as the samples, including filtration at the time of media preparation, subsampling in the ‘non-clean’ environment of the culture experiments, and exposure to the same temperature and light conditions as cultures throughout the duration of the experiments. Total procedural blanks were also processed to quantify the extent of background contamination, if any, introduced by labware or handling. The concentrations of Mn, Fe, Co, Cu and Mo in all blanks (n = 12) were below LOD and therefore no blank correction was applied to the elemental concentration data of the culture samples for these elements. However, the average Zn concentration across all 12 blanks is 4.01 ± 0.9 nmol L^−1^ and was subsequently deducted from the Zn concentrations of all samples.

### Statistical analysis

Statistical analysis was performed with R-studio (version 1.3. 1093-1) using packages plyr, dplyr, tidyverse, multcompView, broom, ggplot2 and ggpubr. Open source data from Stamen was used for map creation via R package leaflet (see Supplementary Information S4, online for references). Plotting was performed using either R-studio or OriginPro, Version 2018 (OriginLab Corporation, Northampton, MA, USA). An ANOVA with a TUKEY HSD post-hoc test was used to determine significant differences in maximum growth rate and maximum cell density between treatments. Data for maximum cell numbers in *D. lemmermannii* were log transformed to achieve a normal distribution.

## Results

### Estimation of the bioavailable fraction of trace metals

The bioavailable concentration of trace metals in the base culture medium (Mix 1 ×) was estimated as 0.45 (± 0.02) nmol L^−1^ for Mn, 3.33 (± 0.23) nmol L^−1^ for Fe, 0.04 (± 0.006) nmol L^−1^ for Co, 0.02 (± 0.002) nmol L^−1^ for Cu, 0.05 (± 0.001) nmol L^−1^ for Zn and 0.32 (± 0.03) nmol L^−1^ for Mo using Visual MINTEQ. Furthermore, concentrations of 1.85 nmol L^−1^ for Mn, 18.8 nmol L^−1^ for Fe, 0.028 nmol L^−1^ for Co, 1.38 nmol L^−1^ for Cu and 0.5 nmol L^−1^ for Zn, were estimated to be bound by the ligand EDTA (Supplementary Table S5, online).

### Concentrations of trace metals in culture media

#### Trace metal concentrations in culture media after the addition of phytoplankton cells

**Figure 1:**
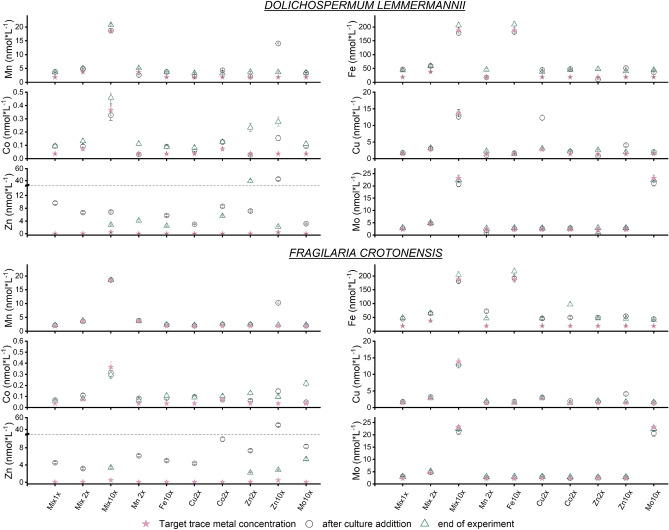
Actual concentrations of trace metals (nmol L^−1^) after culture addition (circles) to growth media and at the end of the experiments (triangles), compared to target trace metal concentrations (purple stars) for (**A**) *Dolichospermum lemmermannii* and (**B**) *Fragilaria crotonensis*. No datapoints are shown when application of a blank correction to Zn concentration yielded a negative result.

Despite efforts to minimise contamination, marked amounts of additional trace metals were added to the medium along with phytoplankton cells at inoculation, as determined by the trace metal concentrations in the base Mix 1 × medium after culture addition (Table 2; Fig. 1, Supplementary Data S6, online). In particular, Zn concentrations were found to be 17- and eightfold higher than the target concentrations for *D. lemmermannii* and *F. crotonensis*, respectively, with distinct differences between species. The respective concentrations of Mn and Co were a factor of 1.7 and 1.3 higher in *D. lemmermannii* with respect to target concentrations, whereas no significant increase was observed for *F. crotonensis* following the addition of culture cells to the medium (Table 2, Supplementary Data S6, online). Iron, Cu and Mo concentrations were also elevated following culture addition, but no significant differences were observed between species.

**Table 2:** Targeted and actual trace metal concentrations after culture addition in the Mix 1 × culture media.

Element	Target concentrations in ‘Mix 1** × **’ (nmol L^−1^)	*Dolichospermum lemmermannii*		*Fragilaria crotonensis*	
		*Actual* concentrations ‘Mix 1** × **’ (nmol L^−1^)	*Percentage difference *(%)	*Actual* concentration ‘Mix 1** × **’ (nmol L^−1^)	*Percentage difference *(%)
Mn	2.3 (± 0.2)	3.82 (± 0.06)	69	2.16(± 0.03)	< 5
Fe	22.1 (± 2.3)	46.7 (± 2.6)	111	44.9(± 2.5)	103
Co	0.072 (± 0.006)	0.095 (± 0.01)	36	0.07 (± 0.01)	< 5
Cu	1.4 (± 0.2)	1.7 (± 0.1)	26	1.8 (± 0.1)	28
Zn	0.55 (± 0.1)	9.7 (± 0.6)	1663	4.6 (± 0.4)	736
Mo	2.3 (± 0.2)	2.7 (± 0.2)	17	3.1 (± 0.2)	34

The corresponding ± 1SD uncertainties are given in brackets.

#### Trace metal concentrations in culture media after phytoplankton growth

**Figure 2:**
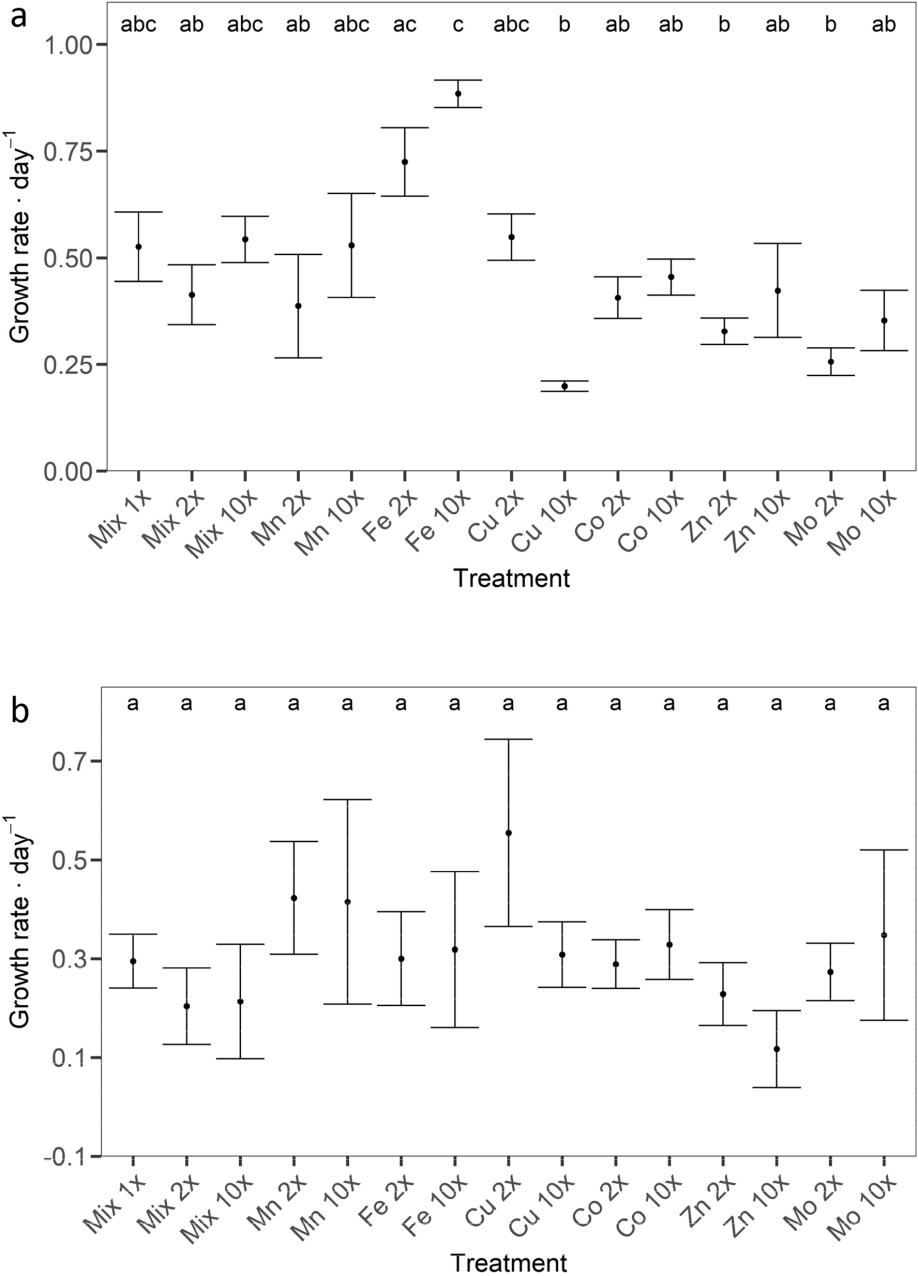
*Dolichospermum lemmermannii* (**A**) and *Fragilaria crotonensis* (**B**) growth rate per day as determined by cell counts. Significant differences between treatments as determined by TUKEY post-hoc test are indicated by the letters at α = 0.05.

For most treatments, there was no marked difference between the ‘start’ and ‘end’ trace metal concentrations (Fig. 2, Supplementary Data S6, online). Manganese was elevated over ‘start’ concentrations at the end of the experiments in treatments Mix 10 × , Mn 2 × and Zn 2 × and was below the ‘start’ concentrations in Zn 10 × for *D. lemmermannii* (Fig. 2). The concentrations of Fe were above ‘start’ concentrations in treatments Mn 2 × and Zn 2 × in *D. lemmermannii* and above ‘start’ concentrations in the Co 2 × treatment, but below ‘start’ concentrations in treatment Mn 2 × for *F. crotonensis.* The ‘end’ concentrations of Co were elevated above ‘start’ concentrations for the treatments Mn 2 × , Zn 2 × and Zn 10 × for *D. lemmermannii* and Fe 10 × , Co 2 × , Zn 2 × and Mo 10 × for *F. crotonensis*. Copper concentrations were lower at the end of the experiment for Zn 10 × and Cu 2 × for *D. lemmermannii* and *F. crotonensis*, respectively. In general, Zn ‘start’ concentrations were elevated above target levels and lower concentrations of Zn relative to target levels were observed for most treatments at the end of the experiment after blank deduction.

### Growth rates and maximum cell density

An average maximum growth rate of 0.46 ± 0.19 day^−1^ was obtained for *D. lemmermannii* across all treatments, with the maximum growth rate of 0.94 day^−1^ recorded in the 10 × Fe treatment. ANOVA showed a significant difference in *D. lemmermannii* growth rates across treatments (*p* < 0.001, Fig. 2a, Supplementary Data S7, online). The post-hoc TUKEY HSD test (Supplementary Data S7 online) showed that growth rates were significantly higher (*p* ≤ 0.01) when *D. lemmermannii* was grown under Fe 10 × concentrations compared to all other treatments (Fig. 2a).

The average maximum growth rate of *F. crotonensis* cultures across all treatments was 0.30 ± 0.19 day^−1^ with the maximum growth rate of 0.75 day^−1^ recorded in the 2 × Cu treatment. There was no significant difference in growth rates between treatments (*p* = 0.66; Fig. 2b, Supplementary Data S8 online).

**Figure 3:**
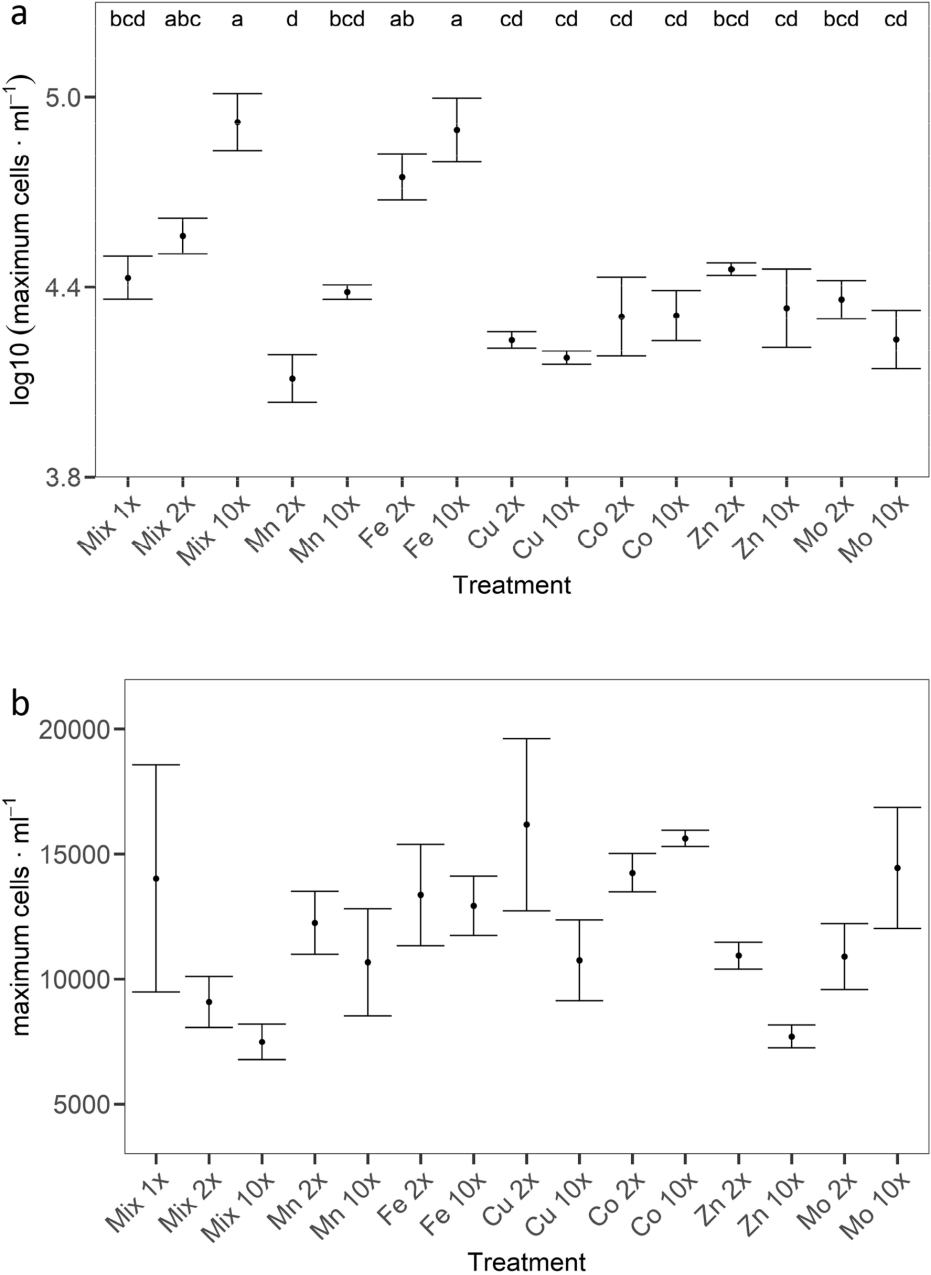
*Dolichospermum lemmermannii* (**A**) and *Fragilaria crotonensis* (**B**) maximum cell density as determined by cell enumeration. Significant differences between treatments as determined by TUKEY post-hoc test are indicated by the letters at α = 0.05. No significant differences were determined for growth rates of *Fragilaria crotonensis*.

Maximum cell densities for *D. lemmermannii* were significantly higher in treatments Mix 10 × , Fe 2 × and Fe 10 × (ANOVA *p* < 0.001) than in all other treatments (Fig. 3a, Supplementary Data S7, online). The maximum cell density at the end of the experiment was 118,762 cells mL^−1^ in treatment Mix 10 × and 114,901 cells mL^−1^ in treatment Fe 10 ×. Conversely, there was no significant effect on *F. crotonensis* cell density from targeted increased trace metal concentrations (ANOVA p = 0.068; Fig. 3b, Supplementary Data S8, online). The highest cell density of 23,109 cells mL^−1^ occurred in treatment Mix 1 × while the lowest cell density was 6065 cells mL^−1^ in treatment Mix 10 ×, both recorded on day 3 of the experiment.

## Discussion

Conducting growth experiments with medium trace metal concentrations in the sub-nanomolar range is challenging due to the high potential for contamination. In this study, although trace metal concentrations in the growth media before culture addition were measured at targeted concentrations, the concentrations of Fe, Mn, Co and Zn in culture media after the addition of cells were elevated. This is problematic when running experiments designed to assess growth responses to low-level (≪ 20 nmol L^−1^) trace metal concentrations.

Possible sources of excess trace metals in the culture media include leaching from cell surfaces, lysis of cells via mechanical stress from pipetting, and trace metals released from cells dying as part of the natural population dynamics in the stock cultures^50^. Furthermore, elevated trace metal concentrations could be an artefact of sample preparation as the rupture of cells may occur during sample filtration. Additionally, trace metals bound within the phycosphere^51,52^, a layer of bacteria similar to the rhizosphere in terrestrial plants, could be introduced to the media upon cell addition. Higher trace metal concentrations measured in media after cyanobacteria stock culture addition, in contrast to diatom addition, might stem from specific traits of cyanobacteria, such as their ability to bind Mn via metallophores^44^ and their ability to potentially synthesize Vitamin B12^53^. Furthermore, cyanobacteria exhibit higher surface to volume ratios than diatoms. Therefore, the size of the phycosphere is proportionally larger in cyanobacteria, allowing for a larger percentage of trace metals per cell volume to be weakly bound to their cell surface. These trace metals could be released upon transfer to the culture media thus increasing the concentration of trace metals in the medium, if a similar biomass of cyanobacteria and diatoms are added at the start of the experiment The differences between the target and measured trace metal concentrations at the start of the experiment, along with the unexpected, but reasonable finding that trace metal concentrations for some elements were higher at the end of the experiment, likely resulting from cell rupture due to filtration, highlight the importance of empirical measurement of trace metal concentrations at multiple stages of the experiment. Despite contamination of trace metal concentrations in the growth media above ‘target’ levels, the growth responses observed for each species are, nevertheless, expected to reflect the actual trace metal concentration in the medium.

Dissolved trace metal concentrations in culture media were expected to be lower at the end of the experiments compared to the ‘start’ concentrations due to uptake by phytoplankton. This was not observed, with Mn and Fe exhibiting an increased concentration at the end of the experiment. This finding may be the result of cell lysis at the end of the growth period releasing intracellular contents back into the culture media as well as possible cell rupture upon sample filtration. The effect of elevated trace metal concentrations due to cell lysis might be more pronounced for cultures of *F. crotonensis* where a decline in cell numbers after an initial growth maximum was observed. Cultures of *D. lemmermannii* were actively growing until the end of the experiment and measured elevated concentrations at the end of the experiment are therefore thought to be an effect of cell rupture at sample filtration. It is possible that cyanobacteria cells burst more readily during filtration than the ‘relatively’ robust diatom cells with their silica frustules. Furthermore, as trace metal uptake rates depend on the bioavailability of elements^54,55^, the uptake rates for the species investigated might be too low at the cell concentrations achieved to see an effect in the dissolved trace metal concentration of the medium.

Elevated concentrations of Zn were most likely introduced by the handling of cultures under standard laboratory conditions, as opposed to clean laboratory conditions. It is well known that Zn contamination can occur easily when working under non-cleanroom conditions, as Zn is ubiquitous in the environment, and is present in dust, hair and a wide range of laboratory materials, such as gloves. This may lead to the introduction of Zn to the samples when manipulating vials and during the pipetting of solutions^46,56^. Therefore, care is required when interpreting the lower concentrations of Zn at the end of experiments as the effects of contamination confound the assessment of biological Zn uptake. In the experiments conducted here, the negative concentration values after blank correction clearly demonstrates the unreliability of the Zn concentration data due to contamination artefacts. The use of trace metal clean workstations when manipulating and subsampling cultures would be advantageous to reduce trace metal contamination to cultures in future studies.

Bioavailable trace metal concentrations in the culture media are estimated to be similar to levels reported in natural systems^37,57^. However, the results of culture experiments must be interpreted with care as growth media, in contrast to natural waters, do not contain multiple ligands, such as humic substances and low-molecular-weight (LMW) ligands, which are active in natural systems. Instead, the culture media contains EDTA, which has at least a ten-fold higher binding affinity for trace metals than many ligands occurring in natural systems, such as DOM (1–40 nmol trace metal∙mg^−1^ C^−1^)^58^ and could therefore lower the effective metal bioavailability^59^. Furthermore, in contrast to natural systems where shifts in pH and temperature influence both the free ion activity and bioavailable fraction of trace metals over time, strictly controlled laboratory experiments can only simulate the physicochemical conditions and the bioavailable fraction of trace metals to a certain extent^49^. Taking all of these factors into account, it is possible that the time-averaged bioavailable fraction is larger in natural lakes than in experimental growth media. Therefore, the concentrations of bioavailable trace metals estimated in this study are predicted to be the minimum bioavailable concentration needed for positive growth responses.

Many growth experiments performed on oceanic phytoplankton have worked with concentrations of trace elements comparable to this study^27,60^. In contrast, most culture studies performed with lake phytoplankton have used trace metal concentrations far in excess of those in this study^30^. A common aspect of all of these studies is the observed increase of growth under elevated Fe concentrations, independent of the phytoplankton species^19,30,61^. Studies performed with cyanobacteria, green algae and diatoms indicate a higher demand for Fe by cyanobacteria^62,63^. In this study, elevated concentrations of all trace elements combined (Mix 10 ×) and Fe alone (Fe 2 × and Fe 10 ×) had the largest effect on the maximum growth rate and maximum cell density of *D. lemmermannii.* Conversely, elevated concentrations of all elements combined (Mix 2 × and Mix 10 ×) and Fe alone (Fe 2 × and Fe 10 ×) yielded no growth response in cultures of *F. crotonensis*, when compared to growth responses of other treatments*.*

A possible explanation for the observed differences in growth rates and maximum cell density between the two species investigated in this study could be that diatom growth is not limited by the trace metal concentrations supplied. In contrast, cyanobacteria were limited by the low Fe concentrations supplied, as indicated by increased growth in treatments of higher Fe concentrations*.* Across all treatments, no significant difference in mean growth rates was observed between the diatom and cyanobacteria species. The maximum growth rates for both species observed in this study are similar to growth rates observed in other studies that report maximum growth rates of 1.08^36^ and 1.25^64^ day^−1^ for *D. lemmermanii* and of 0.4^65^, 0.5^66^ and 0.6–0.8^67,68^ day^−1^ for *F. crotonensis* under non-limited conditions. Generally lower growth rates are associated with limitation. Thus, maximum growth rates of *D. lemmermannii* in the Fe 2 × and Fe 10 × treatments indicate a higher Fe demand for growth of *D. lemmermannii*, and potential Fe limitation at lower concentrations. Conversely, similar maximum growth rates of *F. crotonensis* across all treatments indicate that growth was not affected by the trace metal concentrations tested.

The observed differences in growth response between the two taxa could also be partly due to differences in cell size and metal acquisition processes and requirements. The larger surface to volume ratios of cyanobacteria allow for more trace metal binding sites. Cyanobacteria can also produce metallophores which might make more of the Fe bound by EDTA bioavailable^44^. Therefore, even the highest concentrations of Fe in growth media may not have been as accessible to *F. crotonensis* as to *D. lemmermannii*. However, recent studies have shown siderophore production in a marine diatom^69^ and similar Fe accumulation mechanisms might exist in some freshwater diatoms, however it is not currently known if these are present in *F. crotonensis.* It is also possible that different diatom species would show varying responses to elevated Fe concentrations^70^. Species of the genus *Aulacoseira* or *Asterionella,* which are also abundant in Lake Taupō, would be ideal candidates to test this hypothesis.

A further possibility is that, the diatoms growing in Lake Taupō could have become adapted to lower trace metal concentrations, as observed for other diatom species in low Fe environments, which have lowered the Fe demand of their photosynthesis complex twofold to threefold, compared to species growing under Fe-replete conditions^70,71^. Conversely, the Fe demand of cyanobacteria is potentially higher due to elevated Fe requirements of the photosynthesis complex and nitrogen fixation enzymes^72,73^. This elevated requirement could limit cyanobacteria growth under the lowest Fe concentrations tested.

Physical factors may have also affected the growth patterns of the diatom species tested. For example, *F. crotonensis* is highly abundant in Lake Taupō and other lakes during times of lake mixing, and during times of strong water movement^41^. In the present study, physical disturbance only occurred when the culture bottles were shaken, every 2–3 days prior to subsampling, potentially prohibiting diatom growth.

Future studies should consider growing cultures in low trace metal treatments for a longer time prior to starting the experiment with cells maintained in the exponential growth phase until a consistent growth rate is attained after transfer to a new medium. This would ensure the cells are fully acclimated to low metal conditions. Conducting experiments for a longer time may also be beneficial to observe long term trends of trace metal limitation on algal growth.

The results of this study, and another culture study on cyanobacteria species obtained from Lake Taupō^36^, indicate that cyanobacteria growth in Lake Taupō is likely Fe-limited. Our results also indicate that growth of *F. crotonensis* is potentially not limited at ambient concentrations of trace metals in Lake Taupō. This is an important finding as it shows that any increase in trace metal concentrations in the lake would likely favour cyanobacteria growth.

## Conclusions

Our study strengthens the findings of previous investigations^30,36,61^ and highlights the importance of Fe for cyanobacteria growth and bloom formation. The results suggest that even small increases, twofold in this study, of dissolved and bioavailable Fe concentrations might lead to more frequent and severe CHABs in oligotrophic lakes and may be especially significant in Lake Taupō*.* No effect of elevated trace metal concentrations on growth rates and maximum cell densities of *F. crotonensis* was observed. Therefore, *F. crotonensis* is growing at, or close to, its maximum capacity under ambient trace metal concentrations in Lake Taupō. Future lake management strategies should consider the influence of anthropogenic Fe sources, especially fertilizers, which may reach the lake via land run-off as additional Fe in lake waters may favour harmful cyanobacteria growth. The results of this study also demonstrate the importance of trace metal clean working procedures throughout the entire culturing experiment, in order to minimize the impact of trace metal contaminants and expand the utility of freshwater phytoplankton growth experiments to ultra-low trace metal concentrations at and below the nanomolar range. In addition, the implementation of trace metal concentration monitoring by ICP-MS at different stages of the culturing experiments is advantageous to determine the true trace metal concentrations in the cultures rather than relying on expected concentrations that may be incorrect and skew the accuracy of the experimental findings.

## Supplementary Information

Supplementary Information 1.

Supplementary Information 2.

Supplementary Information 3.

Supplementary Information 4.

Supplementary Information 5.

Supplementary Information 6.

Supplementary Information 7.

Supplementary Information 8.
