# Supplementary material for: Growth at the limits: comparing trace metal limitation of a freshwater cyanobacterium (Dolichospermum lemmermannii) and a freshwater diatom (Fragilaria crotonensis)
Source: Sci Rep. 2022 Jan 10;12:467. doi: 10.1038/s41598-021-04533-9 (PMC8748459; doi:10.1038/s41598-021-04533-9)
Supplement: Supplementary file 1 — Supplementary Information 1. [file 41598_2021_4533_MOESM1_ESM.pdf]

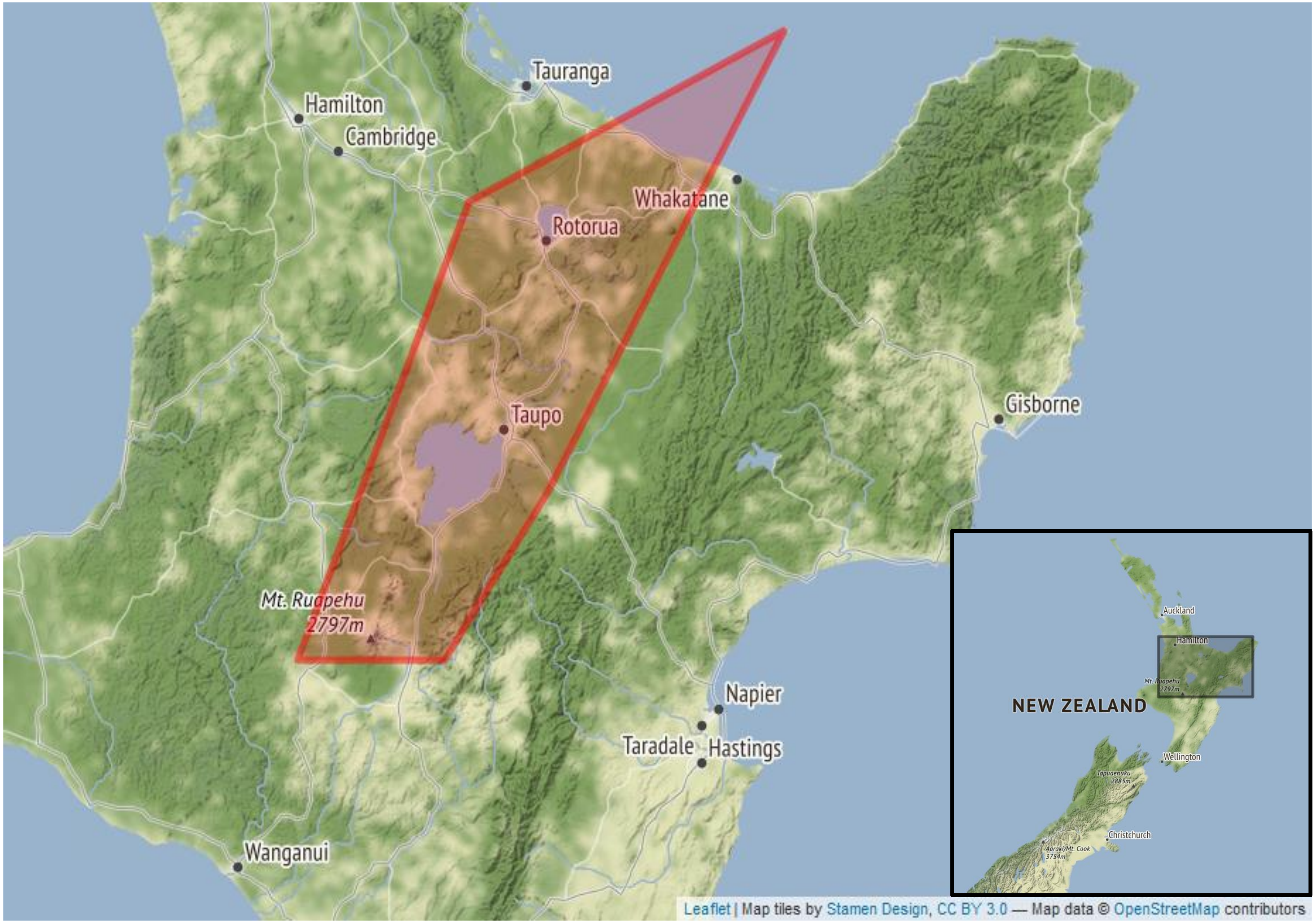

Supplementary Figure S1: Map of the North Island of New Zealand with the spatial extent of the TVZ shown in red.
