# Supplementary material for: Growth at the limits: comparing trace metal limitation of a freshwater cyanobacterium (Dolichospermum lemmermannii) and a freshwater diatom (Fragilaria crotonensis)
Source: Sci Rep. 2022 Jan 10;12:467. doi: 10.1038/s41598-021-04533-9 (PMC8748459; doi:10.1038/s41598-021-04533-9)
Supplement: Supplementary file 3 — Supplementary Information 3. [file 41598_2021_4533_MOESM3_ESM.pdf]

Supplementary Information S3: Additional information on Quadrupole Inductively Coupled Plasma Mass Spectrometry run specific settings.

Manufacturer's recommended general-purpose plasma mode for stability and robustness was used for analysis and the instrument auto tune function was used to maximise sensitivity and minimise interferences.

The acquisition method used 2 different gas modes for the octopole reaction cell.

Gas modes used for analysis: H2 mode for Fe56 and Se78

Helium mode for all other elements collected

All samples calibration standards, blanks and control samples were introduced to the instrument in 2% v/v HNO3.

A cocktail of 7 internal standards containing Be, Sc, Ge, Rh, In, Tb and Bi were added online to correct for matrix effects and instrumental drift.

Calibration standards were prepared from NIST traceable Agilent multi element standards in 2% v/v HNO3.

Calibration curves consisted of a calibration blank and at least 3 calibration levels.

Calibration was verified over the course of the run by running a continuing calibration verification (CCV) standard every 20 samples.

Instrument Settings:

Plasma Parameters

Plasma Mode: General Purpose

Nebulizer Gas: 1.08L/min

Plasma Gas: 15.0L/min

Auxiliary Gas: 0.90L/min

RF Power: 1550w

RF Matching: 1.80V

Sample Depth: 10.0mm

Nebulizer Pump: 0.10rps

S/C Temp: 2degC

Cell Parameters

H2: 6.0ml/min

He: 4.3ml/min

Oxides/Doubly Charged Ratio

Oxides:	0.505%
Doubly Charged:	1.1%

Sample introduction system consisted of an Agilent SPS 4 Auto-sampler, Agilent Integrated Sample Introduction System (ISIS 3) (enabling discrete sampling, faster sample uptake and washout), glass concentric MicroMist nebuliser, quartz spray chamber, quartz torch with 2.5mm diameter injector and Ni interface cones.
