# Supplementary material for: Growth at the limits: comparing trace metal limitation of a freshwater cyanobacterium (Dolichospermum lemmermannii) and a freshwater diatom (Fragilaria crotonensis)
Source: Sci Rep. 2022 Jan 10;12:467. doi: 10.1038/s41598-021-04533-9 (PMC8748459; doi:10.1038/s41598-021-04533-9)
Supplement: Supplementary file 4 — Supplementary Information 4. [file 41598_2021_4533_MOESM4_ESM.pdf]

Supplementary Information S8: References to R-packages used.

Statistical analysis was performed with R-studio (version 1.3. 1093-1) using packages plyr[1], dplyr[2], tidyverse[3], multcompView[4], broom[5], ggplot2[6] and ggpubr[7]. Open source data from stamen was used for map creation via R package leaflet[8].

1. Wickham, H. The split-apply-combine strategy for data analysis. *J. Stat. Softw.* **40**, 1–29 (2011).

2. Hadley Wickham, Romain François, Lionel Henry & Kirill Müller. A grammar of data manipulation [R package dplyr version 1.0.0]. *Media* (2020).

3. Wickham, H. *et al.* Welcome to the Tidyverse. *J. Open Source Softw.* **4**, 1686 (2019).

4. Graves, S., Piepho, H.-P. & Selzer, L. multcompView: Visualizations of paired comparisons. R package version 0.1-8. (2019).

5. Robinson, D., Hayes, A. & Couch, S. Broom: Convert statistical objects into tidy tibbles. *R Packag. version 0.7. 0. https//CRAN. R-project. org/package= broom* (2020).

6. Wickham, H. Elegant graphics for data analysis: ggplot2. in *Applied Spatial Data Analysis with R* 21–54 (2008).

7. Kassambara, A. ggplot2. *Based Publ. Ready Plots [R Packag. ggpubr version 0.2. 5]* (2020).

8. Cheng, J., Karambelkar, B. & Xie, Y. leaflet: Create interactive web maps with the JavaScript'Leaflet'Library. R package version 2.0. 3. (2019).
